# Transcription-related mutations and GC content drive variation in nucleotide substitution rates across the genomes of *Arabidopsis thaliana *and *Arabidopsis lyrata*

**DOI:** 10.1186/1471-2148-7-66

**Published:** 2007-04-23

**Authors:** Leah J DeRose-Wilson, Brandon S Gaut

**Affiliations:** 1 Dept. of Ecology and Evolutionary Biology, U.C. Irvine, Irvine, CA 92697, USA

## Abstract

**Background:**

There has been remarkably little study of nucleotide substitution rate variation among plant nuclear genes, in part because orthology is difficult to establish. Orthology is even more problematic for intergenic regions of plant nuclear genomes, because plant genomes generally harbor a wealth of repetitive DNA. In theory orthologous intergenic data is valuable for studying rate variation because nucleotide substitutions in these regions should be under little selective constraint compared to coding regions. As a result, evolutionary rates in intergenic regions may more accurately reflect genomic features, like recombination and GC content, that contribute to nucleotide substitution.

**Results:**

We generated a set of 66 intergenic sequences in *Arabidopsis lyrata*, a close relative of *Arabidopsis thaliana*. The intergenic regions included transposable element (TE) remnants and regions flanking the TEs. We verified orthology of these amplified regions both by comparison of existing *A. lyrata – A. thaliana *genetic maps and by using molecular features. We compared substitution rates among the 66 intergenic loci, which exhibit ~5-fold rate variation, and compared intergenic rates to a set of 64 orthologous coding sequences. Our chief observations were that the average rate of nucleotide substitution is slower in intergenic regions than in synonymous sites, that rate variation in both intergenic and coding regions correlate with GC content, that GC content alone is not sufficient to explain differences in rates between intergenic and coding regions, and that rates of evolution in intergenic regions correlate negatively with gene density.

**Conclusion:**

Our observations indicated that mutation rates vary among genomics regions as a function of base composition, suggesting that previous observations of "selective constraint" on non-coding regions could more accurately be attributed to a GC effect instead of selection. The negative correlation between nucleotide substitution rate and gene density provides a potential neutral explanation for a previously documented correlation between gene density and polymorphism levels within *A. thaliana*. Finally, we discuss potential forces that could contribute to rapid synonymous rates, and provide evidence to suggest that transcription-related mutation contributes to rate differences between intergenic and synonymous sites.

## Background

The primary processes that contribute to nucleotide substitution rates are mutation, selection, and population history, but their relative contributions can vary substantially among genes and genomic regions. For example, selection varies across genes as a consequence of protein function and gene expression patterns [1]. Similarly, mutation rates vary across genomic regions as a function of base composition and recombination rate [2,3]. Population history may be major determinant of substitution rate in the presence of selection, but should not be a factor in the absence of selection [4].

To date, our understanding about the evolutionary forces that contribute to nucleotide substitution rates has been based primarily on the study of coding regions. Inferences based on coding data reflect, in large part, the action of selection. An obvious example is variation in nonsynonymous substitution rates among genes, which is determined primarily by differential selective constraint. A subtler example is substitution rates at third codon positions, which are a function of both mutation and selection on codon usage. Highly biased genes evolve more slowly at synonymous sites [5-7], but synonymous substitution rates are also correlated with GC content [8-12]. The important point is that it can be difficult to disentangle the contribution of selection and mutation to rate variation among coding regions.

In contrast, non-coding regions should be under little selective constraint, and thus nucleotide substitution in these regions should be governed primarily by neutral processes like mutation. Studying non-coding regions can be difficult in practice, however, because orthology is not always clear. One way to identify orthologous non-coding regions is to compare, for example, non-coding regions that are 5' and 3' to orthologous genes [13]. The problem is that these regions are also expected to be enriched for functional elements, like promoter and enhancer sequences, and thus potentially under strong selection. Another way to identify orthologous regions is to compare non-coding regions among species via similarity (e.g., BLAST) searches, but this approach is also likely to enrich for slow-evolving regions under selective constraint. Indeed, selective constraint on non-coding regions may be more pervasive in eukaryote genomes than previously assumed [14]. For example, there is compelling evidence from *Drosophila *and several mammalian species that some non-coding regions evolve more slowly than synonymous sites [15], presumably due to selective constraint on non-coding nucleotide substitutions [13,14,16]. Thus, if non-coding data are not chosen carefully, it can be as difficult to disentangle the relative contribution of mutation and selection for non-coding data as it is for coding data.

To date, there have been few studies comparing evolutionary rates among plant nuclear genes [17,18]. The dearth of studies reflects, in part, difficulties substantiating orthology relationships, which are complicated because plant genomes contain more duplicated genomic regions than animal genomes [19]. This orthology problem is magnified for non-coding regions, which may evolve and rearrange more rapidly than coding regions. As a result, rates and patterns of sequence evolution among plant non-coding regions have not been characterized in any detail.

Here we contrast nucleotide substitution rates between orthologous coding and intergenic regions of *Arabidopsis thaliana *and *Arabidopsis lyrata*, two plant species that diverged ~5 million years ago [20]. Their genomes are largely collinear, but they differ in chromosome number (*A. lyrata *has eight chromosomes while *A. thaliana *has five; [21]), in DNA content (the *A. lyrata *genome contains ~1.4x more DNA than that of *A. thaliana*; [22]), and by several translocations [23,24]. The two species also differ in population history; *A. thaliana *is predominantly selfing, while *A. lyrata *is an obligate outcrosser. These differences should affect differences in patterns and rates of molecular evolution between species, but the expected differences are not readily apparent [17], perhaps because *A. thaliana *only recently shifted to a selfing mating system [25].

To study rate variation in intergenic regions, we have generated sequence data in *A. lyrata *using PCR primers that flank remnants of transposable elements (TEs) in *A. thaliana*. We reason that these regions are non-functional by virtue of TE insertion, and thus comprise a data set that should be relatively free of selective constraint. We verify their orthology both with comparative maps of the two species and by their molecular features. The intergenic data are contrasted to a second data set consisting of large (> 400 bp) exonic sequences from *A. lyrata *and *A. thaliana*. With these two data sets, we address several questions about *Arabidopsis *nucleotide substitution rates, such as: *i*) do intergenic sequences evolve at rates similar to synonymous sites in coding data? *ii*) do any genomic features, like GC content or recombination, correlate with nucleotide substitution rate variation among loci? *iii*) what can be inferred about the relative contribution of mutation and selection to nucleotide substitution? and *iv*) do intergenic regions provide any hints to the mechanisms that contribute to genome size differences between *A. lyrata *and *A. thaliana*?

## Results

### Isolation and location of orthologous intergenic regions

We identified TE remnants in the *A. thaliana *genome and designed PCR primers to flank 576 of these remnants. Three primers were designed: two flanked the TE remnant, and a third (internal) primer was specific to the TE. We attempted amplification in *A. lyrata *with two separate PCR reactions for each of the 576 intergenic regions. The first used the two flanking primers, and the second used one flanking primer with the internal TE-specific primer. These two primer sets were also applied to *A. thaliana *ecotype Colombia as a positive control (data not shown). The intergenic regions were distributed across the *A. thaliana *genome and ranged in size from 200–2000 bases.

Of 576 attempted *A. lyrata *PCR amplifications, 34% (198 of 576) were successful. Amplifications were considered a success when either of two cases occurred. In case one, the flanking primers amplified a band near the expected size and the internal primer also amplified a band of the expected size. The second successful case was when the flanking primers amplified a band consistent in size with the absence of the TE, and the internal primer failed to amplify any band. PCR failure was usually a failure of both primer combinations, but ~10% of PCR "failure" was unexpected PCR results. Examples include patterns in which flanking primers amplified a band greatly different than the expected size or in which flanking primers amplified a small band consistent with the loss of the TE but amplification with the internal "TE" primer was successful.

In order to understand sampling biases associated with data generation, we mapped the location of PCR successes and failures along the genome to determine whether successful PCR amplifications were clustered with respect to their relative positions on the *A. thaliana *genome. We coded successful and unsuccessful PCRs as 1's and 0's, respectively, and created a binary string to represent the linear order of PCR results. We then calculated the variance in the number of zeros (failed PCRs) bounded by ones (successful PCRs) or the end of chromosomes. The observed variance in our data was compared to a distribution of variances based on 100,000 random shufflings of the binary string. The observed variance was much greater than 0.74% of simulated variances, indicating that successful PCR amplifications were significantly clustered (*p *= 0.0074). This clustering was related, in part, to gene density. We found that attempted amplifications were more likely to be successful in low gene density regions of *A. thaliana *(t-test; *p *= 0.027), when we calculated gene density in a window of 0.50 Mb around the *A. thaliana *TE. Consistent with this observation, all attempted amplifications were successful in regions with gene density lower than 90 annotated genes per 0.50 Mb, although there were only 5 amplifications in regions with density this low [see Additional File 1]. This effect seemed to be relatively local, however, because larger 1.0 Mb windows retained the basic trend but the trend was no longer significant (*p *= 0.12).

Many of the amplicons were small, presumably due to the absence of the TE in *A. lyrata*. We cloned and sequenced a subset of 80 amplicons, yielding 66 alignable sequences. In order to help establish orthology, we examined the position of these 66 amplicons with respect to comparative maps of *A. thaliana *and *A. lyrata *[23,24]. We mapped each amplicon to the *A. thaliana *genome and found its place between markers that had been mapped on *A. lyrata*. We considered an amplicon as belonging to a collinear region of the genome if the two markers flanking the marker were also neighboring markers in *A. lyrata*. By this criterion, we could assign 45 of 66 (68%) to unambiguously collinear regions. Another 20 were ambiguous either because they had only one flanking marker (i.e., they were at the end of a chromosome) or because a third marker had been rearranged between the two markers on the *A. lyrata *map. Only one amplicon was clearly in a non-collinear region. Although the resolution of comparative *A. thaliana-A. lyrata *maps is limited and the possibility of paralogy due to segmental duplication cannot be dismissed entirely, most (68%) of our intergenic regions were orthologous by the criterion of collinearity.

The DNA sequence size of our 66 intergenic regions ranged from 218–1288 bp, with an average length of 732 bp [see Additional File 1]. None of the sequences had extensive open reading frames; the longest ORF was 111 bp. Based on the Arabidopsis small RNA project [26] targetfinder, there were also no obvious conserved small RNAs present in the sequences. Of the 66 regions, 49 *A. lyrata *sequences contained a homolog of the TE remnant found in *A. thaliana*. These 49 sequences were alignable both in the TE remnant and in regions flanking the TE remnant, providing further molecular evidence for orthology. The remaining 17 *A. lyrata *sequences did not contain the TE remnant, but the sequences were homologous to the regions flanking the *A. thaliana *TE. Altogether, 91% (60 of 66) of our amplicons were either in a collinear region or contained the TE remnant with associated flanking regions, providing strong evidence that our amplicons are orthologs.

### Substitution rates in intergenic vs. synonymous sites

**Figure 1:**
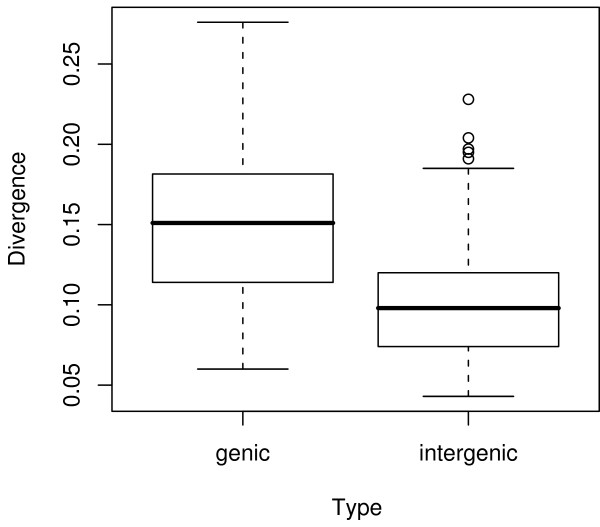
A box plot of genetic distances in the two sequence classes: intergenic and coding. The box represents the interquartile range, with the lines extending the range of the data. Points outside the range are mild outliers, with values greater then 1.5 X the upper bound of the interquartile range.

Given orthologous sequences, we estimated evolutionary distances for intergenic loci. We report distances based on the K2P model (*d*_K2P_) [see Additional File 1], but all results and analyses are qualitatively identical using the general time-reversible model (data not shown). The average *d*_K2P _(d¯
 MathType@MTEF@5@5@+=feaafiart1ev1aaatCvAUfKttLearuWrP9MDH5MBPbIqV92AaeXatLxBI9gBaebbnrfifHhDYfgasaacH8akY=wiFfYdH8Gipec8Eeeu0xXdbba9frFj0=OqFfea0dXdd9vqai=hGuQ8kuc9pgc9s8qqaq=dirpe0xb9q8qiLsFr0=vr0=vr0dc8meaabaqaciaacaGaaeqabaqabeGadaaakeaacuWGKbazgaqeaaaa@2E15@_*K*2*P*_) estimate over all 66 regions was 0.105 substitutions per site (Figure 1). The *d*_K2P _estimates ranged ~5-fold from 0.042 to 0.228 substitutions per site. These numbers of course report the distances as if they were sampled from a single distribution, and *a priori *it might be thought (for example) that sequences with TEs evolve at different rates than those without. There is, however, no evidence to this effect (t-test, *p *= 0.57; Mann-Whitney U, *p *= 0.91). Similarly, we examined whether evolutionary rates differed by collinearity, reasoning that paralogous sequences could be represented in the group for which collinearity was ambiguous. There was no difference between groups based on collinearity (t-test, *p *= 0.81; Mann-Whitney U, *p *= 0.34). Furthermore, the six amplicons for which there was no evidence for orthology from either TE presence or collinearity fell well within the extremes of the *d*_K2P _range (*d*_K2P _ranges from 0.074 to 0.120 for these six; [see Additional File 1]). Because there is no obvious evidence for rate classes based on TE presence or collinearity, we treated the 66 intergenic sequences as a single group.

In addition to our non-coding data set of 66 sequences, we generated an additional set of 64 large (400–700 bp) exons. These exons were distributed throughout the genome [see Additional File 2]. Forty of these 64 genes are located in non-ambiguously collinear regions between *A. thaliana *and *A. lyrata*. Of the remaining 24, 23 had ambiguous collinearity and one was in a non-collinear region. We calculated synonymous distances (*d*_s_) between sequences for all 64 loci. The 24 genes with uncertain collinearity did not differ from the remaining 40 loci either as group (t-test, *p *= 0.80; Mann-Whitney U, *p *= 0.85), but two of these exons had the highest *d*_s _values in our sample, with one value > 1.5-fold higher than that of any other exon [see Additional File 2]. We chose to remove these two loci from further analyses because they may represent paralogs, although none of our overall results vary with their inclusion. Based on the remaining 62 exons, mean estimates of *d*_s_(d¯
 MathType@MTEF@5@5@+=feaafiart1ev1aaatCvAUfKttLearuWrP9MDH5MBPbIqV92AaeXatLxBI9gBaebbnrfifHhDYfgasaacH8akY=wiFfYdH8Gipec8Eeeu0xXdbba9frFj0=OqFfea0dXdd9vqai=hGuQ8kuc9pgc9s8qqaq=dirpe0xb9q8qiLsFr0=vr0=vr0dc8meaabaqaciaacaGaaeqabaqabeGadaaakeaacuWGKbazgaqeaaaa@2E15@_s_) were 0.148 substitutions per synonymous site, with a range from 0.060 to 0.259 (Figure 1).

Our principle goal is to compare substitution patterns and rates between coding and intergenic regions. Loci within the two data types varied ~5-fold in genetic distance. The coefficients of variation, which was 0.41 for intergenic data and 0.33 for coding data, indicate that distances vary to similar degrees for the two data types. However, the mean genetic distance differed between the two classes (Figure 1), with synonymous sites evolving more rapidly than non-coding sites (t-test, *p *< 0.001). Mean distances can be used to estimate nucleotide substitution rates. Assuming that the two lineages diverged roughly 5 million years ago [20], the mean substitution rate for synonymous sites was 1.55 × 10^-8 ^substitutions per site per year. In contrast, the mean non-coding rate was only two-thirds of that value, at 1.05 × 10^-8 ^substitutions per site per year (Figure 1).

### Genomic correlates with substitution rates

Our results indicate that mean genetic distances vary between data types and also that genetic distances vary among loci. We also sought to determine whether genetic distances are related to genomic features such as GC content and recombination rate. We expect that such relationships will be clearer in intergenic data, because the patterns are less likely to be complicated by selective forces.

**Figure 2:**
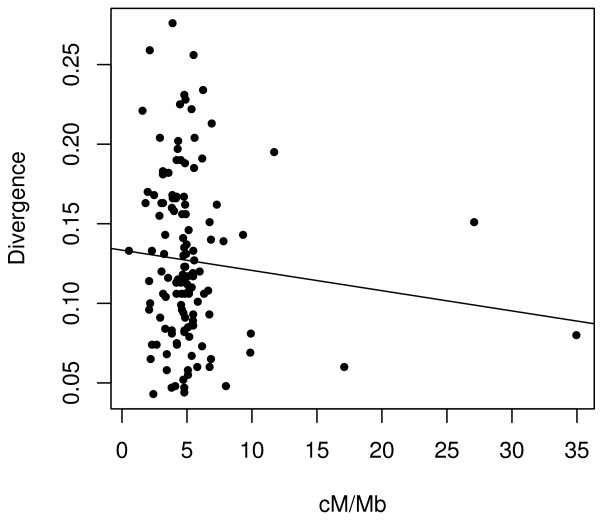
The correlation between recombination rate (x-axis) and genetic distance is not significant for combined coding and non-coding data (*r *= -0.10). Filled circles represent coding loci, and empty circles are non-coding loci.

Recombination may contribute to rate variation among loci by introducing mutations [27,28]. Recombination rates have been estimated for *A. thaliana*, based on comparisons of physical (Mega bases) and genetic (centiMorgan) distances [29,30], but there are no direct estimates of cM/Mb recombination rates for *A. lyrata*. We therefore used *A. thaliana *recombination rates to investigate relationships between recombination rate and genetic distances. There was no strong correlation between recombination rate and genetic distance whether the data were combined (*r *= 0.10; Figure 2) or examined separately as intergenic and genic.

**Figure 3:**
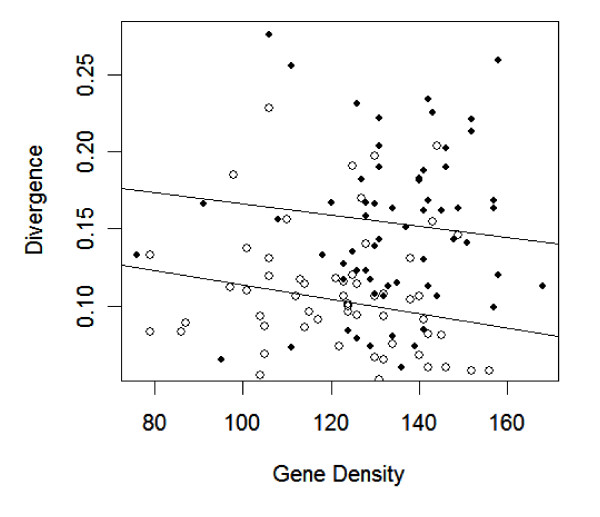
The correlation between gene density, based on the number of genes in a 0.5 Mb window, and divergence is negative for both coding and non-coding data. Filled circles represent coding loci, and empty circles are non-coding loci. The higher regression line is based on coding data.

The lack of correlation between recombination rates and genetic distances parallels a previous study that detected no correlation between recombination rates and *A. thaliana *sequence polymorphism [31]. However, Nordborg et al. (2005) did detect a negative correlation between polymorphism and gene density. Given this observation, we too examined the relationship of genetic distance with gene density. Gene density is negatively correlated with substitution rate in both datasets, although this relationship is only significant in the intergenic data (*r *= -0.33; *p *= 0.086) and not in coding data (*r *= -0.1; *p *= 0.29) (Figure 3). These results were generated with gene density measured as the number of genes in a 0.50 Mb window centered around each intergenic locus; the result is similar, but only borderline significant, with 1.0 Mb windows (*r *= -0.18; *p *= 0.09).

GC content is another factor known to correlate with substitution rate. The GC content of our intergenic sequences did not differ markedly between *A. lyrata *and *A. thaliana *(37.9 and 37.4% GC, respectively). Genic regions also had similar GC content between the two species, but average GC values for coding DNA were 7.3% higher than intergenic regions (44.9% vs. 37.6%, respectively). This contrast also held when only synonymous sites in coding regions were considered (43.1% vs. 37.6%, respectively). The observed transition:transversion ratio was also higher for coding data (1.73) than for intergenic regions (1.36), although statistical support for this trend was borderline (G-test, *p *= 0.09). CpG deamination is a common explanation for elevated transition:transversion ratios in high GC regions and could cause differences in rates between genic and intergenic regions. We thus recalculated divergence, treating all CpG dinucleotides as non-variable in both coding and intergenic data sets. Although there were more CpG sites in genic regions, the CpG disparity alone did not explain the difference in rate between genic and intergenic regions. Genic synonymous divergence rates without CpG sites was 1.22 × 10^-8 ^substitutions per site per year, while intergenic divergence rates were 0.80 × 10^-8 ^substitutions per site per year. These rates are still significantly different from each other (p < 0.001).

**Figure 4:**
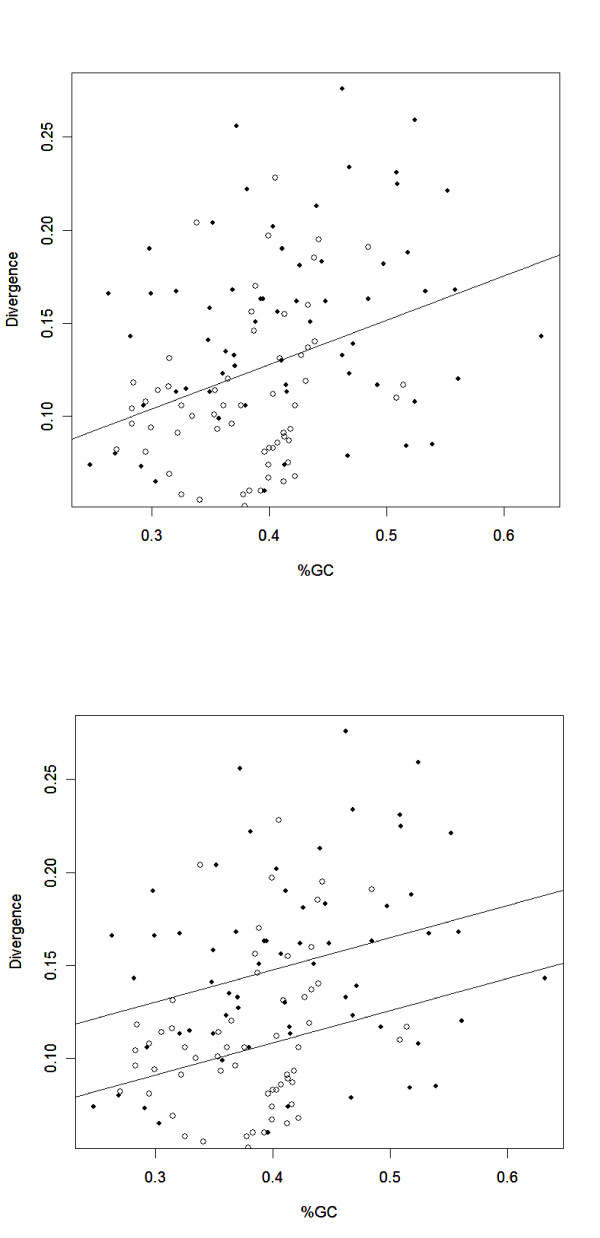
A) The correlation between GC content and genetic distance across both data types (*r *= 0.35; *p *< 0.0001). B) Analysis of covariance with sequence type, GC content and genetic distance. GC content contributes significantly (*p *< 0.003) to the variance in divergence, but there is an additional effect of sequence type on genetic distance that is not accounted for by GC content (*p *< 0.001). For both graphs, filled circles represent coding data and empty circles represent intergenic data.

With combined intergenic and genic data, there was a strong positive correlation between genetic distance and GC content (*r *= 0.35; *p *< 0.0001; Figure 4A). This correlation also held for synonymous sites alone (*r *= 0.33; *p *< 0.001). To better investigate the relationship between GC and genetic distances, we performed an analysis of covariance. The ANCOVA examined the effect of GC on genetic distance, with sequence type as a factor to determine if GC content alone accounted for the differences in genetic distance between our coding and intergenic data (Figure 4B). The ANCOVA model indicated that GC content is a significant predictor for genetic distance (*p *= 0.003), but also that there is an additional effect of sequence type (*p *= 0.001).

### Patterns of indel variation

Intergenic regions do not have constraints on coding frame, and thus accumulate indels. These indels, in turn, may provide insights into processes that contribute to the 1.4× size difference between *A. lyrata *and *A. thaliana *genomes. We analyzed indel size distributions in intergenic data. For these analyses, we ignored sequences in which the remnant TE was present in *A. thaliana *but absent in *A. lyrata*. We also ignored gaps at sequence ends. For the purpose of clarity, we denoted the species with the non-gapped sequence in an alignment as containing an 'insertion'. (In point of fact, one cannot determine whether a gap is due to an insertion or a deletion without the benefit of an outgroup.)

**Figure 5:**
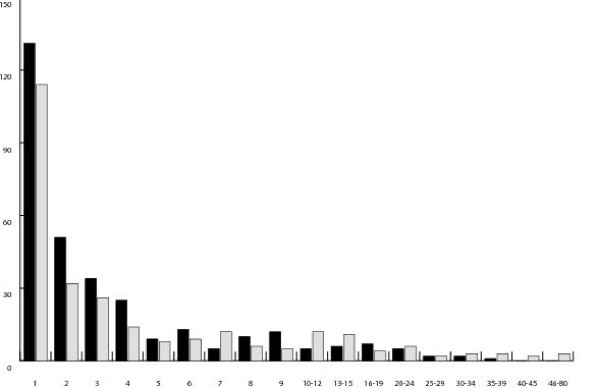
Distribution of insertion sizes in *A. lyrata *and *A. thaliana *intergenic regions. *A. thaliana *insertions are presented in black, *A. lyrata *in grey.

Our intergenic data contained 267 distinct *A. lyrata *insertions totaling 1565 bases. *A. thaliana*, had more distinct insertions (321) but fewer inserted bases (1499). The mean size of insertions was 5.9 bp in *A. lyrata *and 4.7 bp in *A. thaliana*, and did not differ significantly. In general, *A. lyrata *insertions tended to be longer and fewer (Figure 5). Although the distributions of insertion sizes appear somewhat different, with the *A. lyrata *distribution having a longer tail of large insertions and *A. thaliana *having a higher proportion of small insertions, statistical tests comparing the distributions of the samples were not significant (Kolmogorov-Smirnov; *p *= 0.13). Furthermore, a sign test comparing the relative length of *A. thaliana *and *A. lyrata *sequences across loci was not significant (*p *= 0.34), and thus there is no evidence that intergenic regions are systematically longer in *A. lyrata*.

## Discussion

We have generated a sample of orthologous intergenic sequences from *A. lyrata *to compare to *A. thaliana*. These sequences were designed to include remnants of transposable elements (TEs), with the *a priori *thought that these regions are not under strong selection and therefore that the distribution of evolutionary rates provides insight into neutral genomic processes. Nonetheless, our analyses of the genomic location of PCR successes reveal subtle biases in our non-coding data that are important to keep in mind when discussing nucleotide substitution rates. First, the data are not random with respect to genomic location and thus may be biased toward more slowly evolving regions of the genome. Second, they tend to originate from regions of low gene density. This does superficially suggest, however, that they are not enriched for functional elements like enhancers or promoters. Third, most of the sequences originate from regions of genome collinearity. Coupled with molecular features, there is strong evidence that our final data set of 66 non-coding regions represents orthologous DNA features. This set of non-coding sequences represents one of the few – and perhaps only – multi-locus, intergenic divergence data in plants.

We compared our intergenic data to 64 long exons that were sequenced in *A. lyrata*. The primary result is that the mean rate of intergenic nucleotide substitution is two-thirds that of the synonymous coding data, with an absolute rate estimated to be 1.05 × 10^-8 ^substitutions per site per year. This result holds with alternative nucleotide models (see Methods), and thus does not appear to be solely an issue of estimation procedures.

Slower rates in non-coding regions relative to synonymous sites are becoming a surprisingly frequent observation. For example, a recent study of *Drosophila *demonstrated that non-coding DNA evolves considerably slower than synonymous sites in terms of both divergence between species and polymorphism within species [16]. By comparing studies, one can also make the case that pseudogenes [32,33] and introns [34,35] evolve more slowly than synonymous sites in apes and other mammals [13,36-38]. Studies of mammalian intergenic regions have also found slower rates than synonymous sites [35,39,40]. Although most of these studies encompass only a handful of genes, an overall picture of relatively slow non-coding rates is emerging.

Why are non-coding rates slow relative to synonymous rates? One potential reason discussed above (see Introduction) is methodological biases. Our observations were consistent across three different alignment methods and were qualitatively similar when we performed the analyses on synonymous and four-fold degenerate sites using the same K2P model as we applied to intergenic data. Thus, neither model choice nor (mis)alignment appears to contribute substantially to our inferences. Nonetheless, one cannot fully discount sampling biases in the noncoding data, particularly because only 34% of our intergenic PCR amplifications were successful. Some of this failure is attributable to the fact that some TEs, particularly *Basho *elements, do not appear to be shared between the two species and thus could not be amplified in *A. lyrata*. We attempted to evaluate the magnitude and effect of PCR bias by performing a parallel experiment on coding data. We designed three primers to amplify each of the 64 exons, with the outside primers anchored in flanking intronic or UTR regions and an internal primer specific to the exon [see Additional File 2]. To mimic our amplifications of noncoding DNA, we applied the same PCR procedures and same criteria as we applied to noncoding data (see Methods). Although 62 of 64 amplifications were successful in *A. thaliana*, only 25 of 64 (39%) were successful in *A. lyrata*, roughly mimicking the success rate (34%) of noncoding amplifications. The group of exons that were successfully amplified by this method did not differ from "unsuccessful" loci in mean genetic distance (0.154 vs. 0.155, respectively; [see Additional File 2], indicating that PCR success did not heavily bias the inference of genetic distance and substitution rates. If this result is general, then our observation of slow noncoding rates is not solely a function of amplification biases.

A second reason for slow evolutionary rates is selection. Andolfatto (2005) has concluded that non-coding regions of *Drosophila *are under selective constraint and also subject to bouts of adaptive selection. It seems unlikely that this is a general phenomenon, however, particularly for plants. Plant genomes change rapidly in size, in large part due to the accumulation of repetitive DNA [41]. It is thus difficult to imagine that this repetitive DNA is under strong selective constraint. *Arabidopsis *species may be an exception because they have relatively little repetitive DNA for plant genomes. Nonetheless, there is little evidence for selection on non-coding regions that flank coding DNA [42]. Further, the selective interpretation is particularly difficult to argue in this case because the intergenic regions in our study were *a priori *chosen for their apparent lack of function and tend to originate from genomic regions of low gene density. Consistent with the possibility of low functionality, the intergenic data were replete with indel variation. A previous study found that intron lengths from 22 genes differed significantly between these two species [17], suggesting that the 1.4× difference in genome size between *A. lyrata *and *A. thaliana *is due, at least in part, to the accumulation of small sequence changes. Surprisingly, intergenic indel patterns and lengths do not differ substantially between *A. lyrata *and *A. thaliana*, and we find no evidence that differential indel events contribute to differences in genome size.

The third explanation for relatively slow rates of evolution in non-coding DNA is differential mutation rates. Recombination could contribute to this mutational effect, because recombination can cause mutations during the resolution of double-strand breaks [43]. If recombination and mutation are associated processes and most mutations are neutral, then both polymorphism and divergence should be positively correlated with recombination rate. However, we find no correlation between genetic distances and *A. thaliana *recombination rates (Figure 2). Similarly, recombination does not correlate with polymorphism in ~1000 *A. thaliana *gene fragments [31] or 26 *A. lyrata *genes [44]. It is possible, of course, that the lack of correlation between evolutionary rate and recombination is a false negative. A true positive correlation might go undetected if recombination rate estimates are imprecise (see [45]) or if there have been shifts in genomic patterns of recombination between *A. thaliana *and *A. lyrata*. To date, there is little evidence for the latter [23,24,44,46]. Overall, we have no evidence to suggest that recombination rates contribute to the differences in rates between intergenic and genic DNA or to the observed ~5-fold variation in evolutionary rates among loci.

In contrast to recombination rates, there is a strong relationship between genetic distance and GC content in our data (Fig. 3). Substitution rates have long been known to correlate positively with GC content [10,28,47-49], presumably due to higher mutation rates in high GC regions, in part due to CpG effects [8,48,50]. Consistent with these previous observations, the synonymous sites of our coding data have a high average GC content relative to intergenic data, faster evolutionary rates, and a higher proportion of transitional (as opposed to transversional) changes. Interestingly, GC could contribute to some of the non-coding effects observed in *Drosophila*, too. There is a striking difference in GC content between the coding and non-coding regions analyzed by Andolfatto (2005); non-coding sequences have a mean GC content of 43%, compared to 58% GC in coding regions. This disparity alone, rather than selective constraint, may account for the slow rate of divergence in *Drosophila *non-coding DNA. GC content alone cannot explain, however, the skewed ratios of polymorphism to divergence found by Andolfatto (2005), unless there has been a recent shift in GC mutation biases.

Nonetheless, GC content does not provide a complete explanation for evolutionary rate variation in our data. One of the most interesting aspects of genome-wide analyses in *A. thaliana *has been the negative correlation between gene density and polymorphism in *A. thaliana *[31]. This correlation has been interpreted to be the consequence of either selective sweeps or, more likely, background selection [31,51]. We have uncovered a similar negative correlation between gene density and evolutionary rate for intergenic data (Figure 3), providing a potential neutral explanation for the observation – i.e., that mutation rates are higher in regions of low gene density. Note also that, if anything, our intergenic data are biased to low gene density regions of the genome where hitchhiking and background selection should not be particularly strong. The negative correlation between genetic distance and gene density remains borderline significant with partial correlations that also consider GC content (*r *= -0.21, *p *= 0.08). Thus, the gene density effect does not appear to be solely an issue of GC, but the causes of this effect are elusive at this point.

More importantly, there is a "sequence-type" difference between genic and intergenic substitution rates that is not accounted for by differences in GC content (Figure 4B). Differential selection of genomic regions is unlikely to explain the observed differences between sequence types, as we would expect the substitution rate effect to be in the opposite direction (Figure 1). It is possible, though, that mutation varies between intergenic and coding regions. For example, transcription-related mutations could increase synonymous substitution rates in coding regions over and above GC effects. This possibility seems plausible because *A. thaliana *base composition varies as a function of gene expression, suggesting that mutation rates among genes scale with transcription rates [42]. If this hypothesis is correct, then introns should evolve at a rate that is more similar to exons than to intergenic regions. To examine this hypothesis, we aligned the 29 available *A. lyrata *introns from Genbank to their *A. thaliana *ortholog. The mean distance (d¯
 MathType@MTEF@5@5@+=feaafiart1ev1aaatCvAUfKttLearuWrP9MDH5MBPbIqV92AaeXatLxBI9gBaebbnrfifHhDYfgasaacH8akY=wiFfYdH8Gipec8Eeeu0xXdbba9frFj0=OqFfea0dXdd9vqai=hGuQ8kuc9pgc9s8qqaq=dirpe0xb9q8qiLsFr0=vr0=vr0dc8meaabaqaciaacaGaaeqabaqabeGadaaakeaacuWGKbazgaqeaaaa@2E15@_*K*2*P*_) between intron sequences was 0.157 substitutions per site. This distance does not differ significantly from our synonymous divergence estimates (*p *= 0.34), but it is significantly higher than the mean genetic distance at our 66 intergenic loci (*p *= 0.006). Thus, although the data are limited, intronic sequences are consistent with the hypothesis that transcription-related mutation contributes to differential substitution rates between exons and intergenic regions.

## Conclusion

It is clear that GC content is a major determinant of evolutionary rate variation, not only between sequence types (intergenic and coding) but also among loci. GC content may contribute to *A. thaliana *polymorphism, too, because there is a positive, borderline significant correlation (*r *= 0.21; *p *= 0.08) between GC content and SNP polymorphism (as measured by π, the average pair wise difference among a sample of sequences) for 140 polymorphic intergenic loci in the Nordborg *et al*. (2005) data panel of 96 individuals (data not shown). On the other hand, GC content does not fully explain intergenic rate variation; variation also correlates with gene density after correcting for GC content. More importantly, there is a detectable effect of sequence type. The explanation that we deem most reasonable for contributing to the sequence-type effect is transcription-related mutation. Transcription-related mutation predicts a pattern of higher evolutionary rates in transcribed regions, and this prediction is upheld with both exonic and intronic data.

## Methods

### Sequence data

To isolate and sequence orthologous non-coding regions, we started by identifying TEs in the *A. thaliana *genome with a BLASTn search, using a 1e-20 cut-off and no repeat filtering, against the release 4 genome. The TE queries in this search were tabulated from three sources: *i*) TEs described in a previous survey of 17 Mb of the *A. thaliana *genome [52]; *ii*) *A. thaliana *TEs found in TIGR's repeat database; and *iii*) all GenBank ORFs annotated as transposase-related in the *Arabidopsis *genome release 4.0. In this search, we identified 3,079 non-redundant TE sequences ranging in length from 65 bp to 15.8 kb, with a mean length of 1,134 bases.

We designed primers to flank 576 of these TE remnants. Primers were based on *A. thaliana *genomic sequence using primer3 with default parameters. Three primers were designed: two flanked the TE remnant, and a third (internal) primer was specific to the TE. We attempted amplification in *A. lyrata *with two separate PCR reactions for each of the 576 intergenic regions. The first used the two flanking primers, and the second used one flanking primer with the internal TE-specific primer. These two primer sets were also applied to *A. thaliana *ecotype Colombia as a positive control (data not shown). PCR was performed with a 58/51 touchdown protocol with 1 minute denaturing at 95°C, 45 second annealing at 58°C, and 1.5 minute extension at 70°C for 15 cycles, followed by 10 cycles of 1 minute denaturing at 95°C, 45 second annealing at 51°C, and 1.5 minute extension at 70°C, and completed with a 7 min elongation period at 70°C. Plant material for all *A. lyrata *PCR and sequence data was extracted from a single Icelandic individual (provided by S. Wright, York University). DNA extraction employed the DNeasy plant mini kit with the standard protocol.

PCR amplicons were cloned using the pGem-T Easy vector system. Amplicons with multiple bands were gel purified before cloning. In total, 198 regions were successfully amplified in *A. lyrata*. Only a subset of these were cloned and sequenced, however, since many of the amplified regions apparently did not contain TEs in *A. lyrata *and thus the amplified regions were quite small. Additionally some intergenic regions were larger then could be readily cloned. The clones were sequenced using the standard ABI sequencing protocol with BigDye 3.1 terminator kit, and sequences were visualized on an ABI 3100. For most amplicons only a single clone was sequenced. However, a set of eight intergenic regions was cloned and sequenced five times to estimate error from cloning and sequencing. The error rate was estimated to be ~2.5 × 10^-4 ^errors per site. At this rate, error contributed only ~2% uncertainty to our distance estimates between species, and we thus disregarded error in subsequent analyses. Intergenic sequence data were submitted to Genbank [see Additional File 1].

We also amplified and sequenced a set of 64 exons from the same Icelandic accession used to isolate intergenic regions. As explained in Wright et al. (2006), each exon was submitted to a BLAST search [53] against the shotgun genome sequence of *Brassica oleracea*. Homologous *B. oleracea *regions were aligned to *A. thaliana *data to identify conserved regions for primer design. PCR primers were designed with PrimerQuest (Integrated DNA Technologies). Primers and exons were also submitted to a BLAST search against the *A. thaliana *genome to ensure single-copy status. The primers, as well as the list of loci, are provided in Additional File 2 and also described in Wright et al., (in prep). Exon amplifications were based on PCR conditions that included 30 cycles of 30 second denaturing at 95°C, 45 seconds annealing at 55°C, and 1 min extension at 70°C. Amplification products were sequenced directly using ABI BigDye 3.1 and the ABI 3100 automated sequencer. Bases were called using Phred and Phrap. Heterozygotes were resolved with Polyphred [54] and extensive manual trace examination. Only one haplotype was used per locus for analysis. The data are available in Genbank [see Additional File 2].

### Alignments

Nucleotide alignments for both intergenic and coding sequences were generated with ClustalW [55], using default parameters. Indels in the intergenic sequences created some ambiguity in a subset of the alignments. These alignments were inspected manually and in cases where there was a clear resolution the alignments were adjusted manually with Bioedit. Because of our concern about alignment ambiguity bordering indels, we generated a second alignment set for the intergenic data, eliminating eight bases on either side of each indel. We also aligned all intergenic sequences using mcalign2, a program specifically for alignments of intergenic sequences containing indels [56]. This program is optimized for *Drosophila *indel patterns, and parameters for *Arabidopsis *deletions are not known. However, mcalign2 alignments were nearly identical to the original alignments. We also eliminated 14 sequence alignments from analyses either because extensive indels made confident alignments impossible or because a substantial portion of the *A. lyrata *sequence did not appear to be homologous to the *A. thaliana *sequence. Sequence alignments are available from [57].

### Sequence analyses

Genetic distances between *A. lyrata *and *A. thaliana *intergenic sequences were estimated using the Kimura 2-parameter (K2P) model implemented in Mega 2.0. Coding frames for determining synonymous sites was established from *A.thaliana *gene annotations. The Nei-Gojobori method implemented in MEGA2.0 [58] was used to estimate K_S _in the coding sequences. Genetic distances for coding regions were also calculated using only four fold degenerate sites and implementing the K2P model. Changing models did not affect the overall mean divergence estimates, and the original estimates based on the Nei and Gojobori model are presented in all analyses. Recombination rate estimates were based on a previous analysis of *A. thaliana *[30].

## Authors' contributions

LJDW and BSG designed the experiments and analyses and wrote the paper; LJDW performed experimental work and analyses. Both authors read and approved the final manuscript.

## Supplementary Material

Additional file 1 Supplemental information for intergenic sequences. Information, including genetic distance estimates, primer sequences and genbank IDs, for each intergenic sequence analyzed. Click here for file

Additional file 2 Supplemental information for genic sequences. Information, including genetic distance estimates, primer sequences, gene identifiers, genbank IDs and other information for each genic sequence analyzed. Click here for file
